# Design of a Miniaturized Wide-Angle Fisheye Lens Based on Deep Learning and Optimization Techniques

**DOI:** 10.3390/mi13091409

**Published:** 2022-08-27

**Authors:** Chuen-Lin Tien, Chun-Yu Chiang, Wen-Shing Sun

**Affiliations:** 1 Department of Electrical Engineering, Feng Chia University, Taichung 40724, Taiwan; 2 Ph.D. Program of Electrical and Communications Engineering, Feng Chia University, Taichung 40724, Taiwan; 3 Department of Optics and Photonics, National Central University, Chungli 32001, Taiwan

**Keywords:** wide-angle fisheye lens, deep learning algorithm, optimization design, modulation transfer function (MTF)

## Abstract

This paper presents the optimization design of a miniaturized five-element wide-angle fisheye lens using a deep learning algorithm. Zemax optical design software was used to simulate and optimize the wide-angle fisheye lens. A deep learning algorithm helped to find the best combination of different lens materials. We first used six lens elements as an initial configuration to design miniaturized wide-angle fisheye lenses using the optimization process. The optical system components were gradually decreased to five lens elements. Both OKP4HT and polymethyl methacrylate (PMMA) plastic aspheric lenses were selected to replace the second spherical glass lens in the original design. We propose two types of wide-angle fisheye lens designs with four spherical lenses and one aspheric lens. The results for these designs indicated a viewing angle of 174°, a total length of less than 15 mm, a spot size of less than 6 μm, lateral color within ±1 μm, field curvature within ±0.02 mm, and F-θ distortion of ±3.5%. In addition, the MTF value was larger than 0.4 at the spatial frequency of 100 cycles/mm.

## 1. Introduction

Generally, an optical lens system with a field of view (FOV) larger than 120° is called a fisheye lens. Ultra-wide-angle fisheye lenses are refractive optical systems capable of imaging an entire hemisphere or a near-180° field of view onto a flat image plane. Unlike ordinary lenses, miniaturized wide-angle lenses are associated with unavoidable image distortion. A common solution is to correct the captured image by image processing to restore the original image. At present, the fisheye lens is widely used in panoramic photography, monitoring systems, unmanned vehicles, etc.

Wood [1] presented the original fisheye lens structure. He used a pinhole in front of a container filled with water to simulate fisheye viewing in water. Based on the structure described by Wood, Bond [2] proposed a hemisphere lens that performed the same function but used the glass material rather than a water container. A single hemispherical lens was used to replace the water in the design proposed by Wood. In fact, the design by Bond should still be considered a fisheye pinhole camera rather than a fisheye lens. After that, attempts were made to eliminate the aberration found in Bond’s design. Hill [3] used a negative-meniscus lens in the front to guide light into the stop aperture, then added two lenses behind the stop for imaging and aberration control. Bond’s design was further improved by replacing the flat plate with a negative-power meniscus lens. The negative meniscus shape of the first element has become common on all modern fisheye lenses. Beck [4] presented a fisheye lens design using a highly divergent meniscus lens as the first element to produce light into a wide FOV and then used a convergent lens system to project the FOV onto a camera’s image plane. To increase the viewing angle, Shimizu et al. [5] proposed a wide half-field angle—from 90° to 110°—fisheye lens. This wide-angle fisheye lens system includes a forward lens group of negative refractive power and a rearward lens group of positive refractive power. The forward lens group of the fisheye lens system comprises three negative meniscus lenses combined with a biconcave lens and a positive lens. Li et al. [6] proposed a fisheye lens camera and a convex reflective mirror to form a panoramic stereo imaging system. The optical axis of the fisheye lens is aligned with the optical axis of the mirror. The system can generate acceptable 3D reconstruction results within a certain depth range and facilitates a large vertical field of view for monitoring the surrounding environment. In 2016, Sun and Tien et al. [7] reported the relative illumination analysis of an ultra-wide angle lens design with seven lenses. They used F-θ distortion to replace the optical distortion associated with the ultra-wide-angle lens. The ultra-wide angle lens design results showed a total FOV of 160°, F-θ distortion of less than 2%, and relative illumination greater than 83%. In 2017, Yan and Sasian [8] designed a zoom fisheye lens for circular and diagonal fisheye imaging. Compared with the current designs in the market, the zoom fisheye lens has a large aperture while maintaining a simple structure because it uses only 11 lens elements in the two groups. In 2019, Fan and Lu [9] reported a simple design of a fisheye lens using two negative-meniscus lenses and three singlet lenses. All their lenses were made of glass, with a total length of no more than 150 mm; the resultant starting design of the fisheye lens led to a small field curvature, astigmatism, and chromatic aberrations. However, the authors did not discuss the distortion issue in the fisheye lens designs.

The miniaturization of optical lenses is a main theme in the development of optical systems. Novel optical lenses with ultrathin structure and light weight are important for the miniaturization of state-of-the-art optical systems. By replacing bulky conventional lenses, researchers not only succeeded in demonstrating novel metalens designs but also advanced the metalens-based optical systems with ultra-compact dimensions. To achieve the purpose of miniaturization of the wide-angle lens, several studies on metalens have been proposed. The metalens has become a breakthrough technology in the development of miniaturized optical systems due to its outstanding characteristics, such as ultra-thinness and cost-effectiveness [10]. Compared to conventional macro-scale optics manufacturing methods, the micromachining process of metalens is relatively simple and more suitable for mass production. In 2018, Colburn et al. [11] demonstrated a tunable Alvarez metalens system with a large area focal length change for varifocal zoom imaging. The largest focal length range is 6.62 cm at 1550 nm and 32.4 cm at 633 nm. In 2021, Zhang et al. [12] proposed a high-efficiency and ultra-wide angle wavefront control technique for single-chip planar optical elements. They demonstrated its potential in infrared polarization imaging and laser beam scanning, with the largest diffraction-limited imaging (FOV up to 178°) for single-chip planar devices. In 2022, Luo et al. [13] reported a review of recent advances in wide-angle metalenses, including operation principles, design strategies, and applications. Nevertheless, it is still difficult to build a high-performance wide-angle lens, even when using a meta-surface containing sub-wavelength substructures.

In recent years, experts from optical lens design-related fields have invested in the deep learning application of artificial intelligence (AI). Deep learning is a subset of machine learning, in which an artificial neural network (an algorithm inspired by the human brain) learns from a large amount of data. Similar to the way we learn from experience, the deep learning algorithm repeats a task and adjusts it slightly each time to improve the results. Deep learning algorithms also scale the data, but the traditional machine learning saturates. Thus, optical lens design is an area where deep learning can be used. We used the deep learning toolbox in MATLAB, which provides a framework for lens design and selects the suitable lens materials. Next, ZEMAX optical design software was used to optimize the design of a miniaturized wide-angle fisheye lens. In the following sections, the design of a miniaturized wide-angle fisheye lens, the design specifications and results, and the problems involved in tolerance analysis are briefly introduced according to the manufacturing probability with the modulation transfer function (MTF). This paper presents a miniaturized wide-angle fisheye lens system design using an OKP4HT and polymethyl methacrylate (PMMA) aspherical lens to reduce the aberrations and the number of lenses. As mentioned above, many wide-angle fisheye designs and products are commercially available, but most of them are designed with multiple glass lens combinations. In this study, we propose an optimization method using two polymer aspheric lens structures and a deep learning algorithm to achieve a miniaturized wide-angle fisheye design. We also considered the use of fewer lens elements to eliminate the aberrations. Our optical simulation results showed that the proposed lens system can meet the design requirements and has a good performance. The main contribution of this paper is the description of a new design method for a miniaturized five-element wide-angle fisheye lens, which combines a deep learning algorithm and a damped least-square (DLS) optimization technique. The proposed approach can achieve a a wide-angle fisheye lens design with good performance.

## 2. Design Method

The simplest optical system for aberration compensation is a two-lens-group structure. A compact fisheye objective lens consists of six lenses [14]; the first lens element has a convex surface facing the object and a concave surface facing the image plane. The second lens is an aspherical lens and has a concave surface facing the object and a concave surface facing the image. The third and fourth lenses are single lenses. The fifth and sixth lenses combine to form a positive-power doublet lens. To start with, the Gaussian solution was calculated according to the thin lens theory, and the two-lens group structure (a negative lens group and a positive lens group) was determined by a deep learning algorithm.

### 2.1. Wide-Angle Fisheye Lens Design

The two-lens-group structure was first calculated by finding a Gaussian solution based on the thin lens theory. Next, the ZEMAX OpticStudio [15] optical design software package was utilized to optimize the ultra-wide-angle fisheye lenses. The F-θ distortion is a critical problem to be reduced in the optimization process. The F-θ distortion [16,17] is expressed as

(1) $$F\;-\theta \;distortion=\frac{H\prime -H}{H}\times 100\%$$

where *H*′ is the height of a real image, and *H* is the height of the ideal image. *H* can be calculated by

(2) H=f×tanθ

where *f* is the focal length of the lens, and *θ* is the half field of view in radians. 

In our proposed lens design, the second lens was set as an aspheric surface. The equation of aspherical surfaces can be written as

(3) $$s=\frac{c_{v}y^{2}}{1+\sqrt{1-Pc_{v}^{2}y^{2}}}+By^{4}+Cy^{6}+Dy^{8}+Ey^{10}$$

where *s* is the sag of the surface, cv is the vertex curvature of the surface, y is the vertical distance from any point on the surface to the axis of revolution, *P* is the constant term of a conicoid surface, *B*, *C*, *D* and *E* are the high-order coefficients of the aspherical surface. The higher the order of the aspheric coefficient used, the more challenging the lens fabrication. In the optimization process with aspheric coefficients, the order of the aspherical coefficients was used until the 10th order, which contributed little to image quality improvement.

The damped least-squares (DLS) method automatically assigns a damping factor to each parameter in a way that compensates for the relative sensitivity of the variables. Based on the DLS method with good stability, we introduced an optimal enhancement coefficient to achieve faster iteration and more accurate convergence. The optimal enhancement coefficient was used to optimize the damped least-squares method. The advantage of this approach is that it can be used to achieve faster iteration and convergence. The average number of iterations in this method is less than 10, which greatly improves the solving speed. In addition, a merit function (MF) was used to assess the quality of the wide-angle fisheye lens design. The merit function comprises a set of aberrations that need to be corrected or minimized to certain values [18]. This function is a numerical representation of the degree to which an optical system satisfies a specific target set. It is usually impossible to simultaneously set all aberrations in the lens to zero; thus, the choice of the aberrations and the selection of the targets for these aberrations is a critical part of the design process. The value of the merit function is defined by the root sum square of the weighted aberration values:

(4) $$MF=\sqrt{\sum_{i=1}^{n}[W_{i}(X_{i}-T_{i}){]}^{2}}$$

where *MF* is the merit function, *n* is the total number of controlled Seidel aberrations, *W_i_* is the weight applied to the *i*th aberration difference of (*X_i_ − T_i_*), *X_i_* is the controlled Seidel aberration, and *T_i_* is the target value of the lens design. The goal in lens design is to make this function zero. The larger the value, the further the lens is from the desired solution. The computer program selects the controlled aberrations and weightings. The performance of a miniaturized wide-angle fisheye lens is measured by a merit function, and the goal in the optimization stage of the design is to determine the configuration of the wide-angle fisheye lens system for which the merit function is the smallest over the region of interest defined by the constraints. In other words, the objective of the optimization is to obtain the global minimum value of the merit function. The merit function of the ZEMAX software is used to set the curvature, air spacing, glass materials, and coefficients of the aspheric surfaces as variables. The approach for optimizing two kinds of wide-angle fisheye lenses by means of the merit function is described herein. First, the weights and targets of the merit function set the variables such as curvature, air spacing, glass materials, coefficients of the aspheric surfaces, etc. These are changed from time to time as the design proceeds, to force the solution to proceed in a desirable way. When the result meets the expected specification of a miniaturized wide-angle fisheye lens, the optimization process stops. Otherwise, the above steps are repeated until better results are obtained.

**Figure 1:**
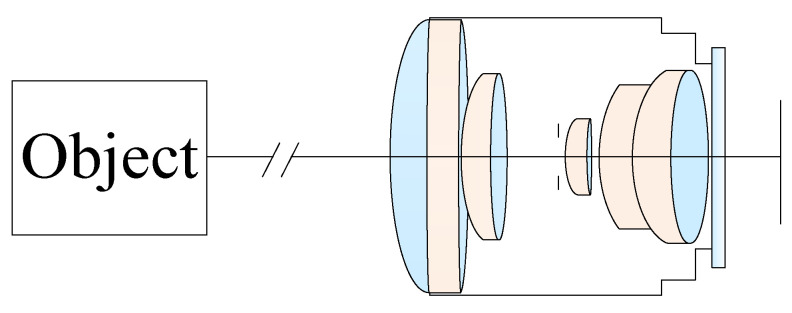
Initial configuration of the miniaturized wide-angle fisheye lens system.

For an optical lens design, the starting point is to choose the symmetrical fisheye lens designed with reference to the relevant US patents [14,19,20,21]. We used the ZEMAX and MATLAB software to simulate and analyze this system. The design specifications of a miniaturized wide-angle fisheye lens are listed in Table 1. In order to accept more light into the lens, the f-number was selected to be 2.8, and the FOV was larger than 170 deg. The first element of the wide-angle lens needs a larger diameter to accept light from an extremely wide angle. The effect focal length (EFL) was larger than 0.6 mm, and the MTF was higher than 30% at a spatial frequency of 100 lp/mm. The field curvature should be less than 0.5 mm, and the spot size was less than 5 μm. The F-theta distortion was less than 5%, and the lateral color was less than 2 μm. The total length of the miniaturized wide-angle fisheye lens was less than 20 mm. To meet the design specifications for the miniaturized wide-angle fisheye lens system, the ZEMAX software was used with merit functions for optimizing the MTF performance and F-theta distortion of the lens system. The initial configuration of the miniaturized wide-angle fisheye lens system is shown in Figure 1. We used the initial design parameters from the patents in the United States of America as design values to form a six-lens-element fisheye system and then decreased the fisheye lens system to a five-element lens. In our design cases, the first, the third, the fourth, and the fifth lenses were spherical lenses, the second component was an aspheric lens (using OKP4HT or PMMA material), and the last element was cover glass for the image sensor. From the technical point of view of optical design, the position of the aspheric lens has great influence on the performance of an optical system [22,23]. To find the most effective position among all the possibilities is one of the important tasks of optical lens designers. In the proposed optical system, selecting the second element as an aspheric lens could effectively eliminate optical aberrations. The last one was a protective cover glass for the image sensors. The cover glass also plays a vital role in optical recording devices to help camera users capture the sharper images they want.

### 2.2. Deep Learning Algorithm 

Due to the discreteness and diversity of the lens materials, it is difficult to deal with them in optical lens design software. In the optimization process, it is usually necessary to allow a continuous optimization of the lens variables, even if only a discrete number of glass materials exist, and then fix them at some point in the process. Although these traditional glass variables help to select approximate lens combinations for a first-order optical layout, there may be other more appropriate methods when the goal is to directly optimize the variables or predict them in machine learning applications. We applied a deep learning (DL) algorithm to process the lens data in the wide-angle fisheye lens design. Deep learning is a subfield of machine learning based on artificial neural networks [24,25,26,27]. The first challenge of applying DL to optical design is that a deep neural network (DNN) model can be trained to predict the response of a given design, but the opposite is not possible. A second challenge in applying DL to optical lens design concerns the model uncertainties involved in DNNs and their implication on the correctness and optimization of the designs. However, this issue has remained unaddressed [28]. It can be solved by a deep learning strategy trained to extrapolate data from the lens design database, which can be used to directly select the lens design starting point from the required first-order specifications of the thin lens. There are potential benefits in borrowing some of the tools used in machine learning and deep learning practice and applying them to a wide-angle lens design. Since a deep neural network is trained to extrapolate data from lens design databases, it can be used to obtain a selection of a wide-angle lens design starting points directly from the desired first-order specifications. The deep learning method trains a deep neural network (DNN) model, which learns the mapping from the required input specifications of all required lens variables [29]. 

**Figure 2:**
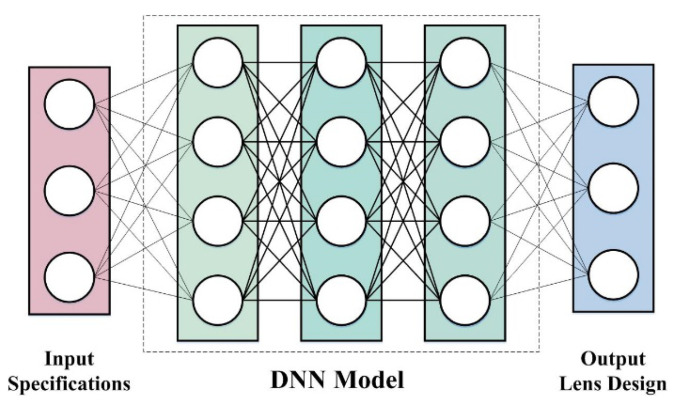
Framework with the training process for the lens design.

The DNN model takes the required first-order specifications (such as lens structure and field of view) as the input and directly infers all the variables required to fully define the lens design, namely, curvature, thickness, refractive index and Abbe number. A single DNN model captures the representation of multiple shot sequences characterized by the sequence of the lens elements. We successfully trained our DNN model on 106 different lens materials. The framework with the training process is illustrated in Figure 2. We also propose a lens selection method from the practice of deep learning and used the ZEMAX software to integrate it into the wide-angle fisheye lens design. Our proposed method is a promising design tool for wide-angle lens optimization.

## 3. Results and Discussion

We present a miniaturized wide-angle fisheye lens system with five lens elements and chose both OKP4HT and PMMA materials for an aspheric lens to replace the second spherical lens. A lens designer wants to know whether the image structure will meet the specifications. The optical transfer function (OTF) provides the most common link between the lens aberration contents and the questions asked by the optical designer. The OTF was used to obtain information about the contrast in the images of specific objects. The basic object used in defining the OTF was a sinusoidal intensity distribution. The MTF is the ratio of the modulation contrasts in the object and image of a sinusoidal pattern. We optimized these two miniaturized fisheye lens designs and present herein the optimization design results; the MTF curve is also discussed. In addition, tolerance analysis is a very important step in optical system design. Since it is impossible to manufacture all optical elements perfectly, tolerances must be specified so that the optical elements can be manufactured within the set tolerances. Tolerances will degrade the design merit function and also affect image quality. Therefore, a well-specified tolerance can maintain a good system performance and also make it easier to manufacture the optical components.

### 3.1. Aspheric Lens Using the OKP4HT Material

**Figure 3:**
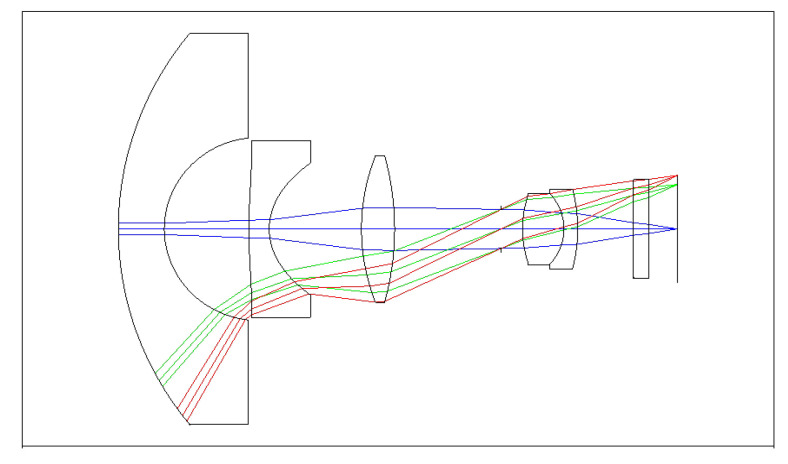
Five-lens structural design of a miniaturized wide-angle fisheye lens using an OKP4HT aspherical lens to replace a glass spherical lens.

**Figure 4:**
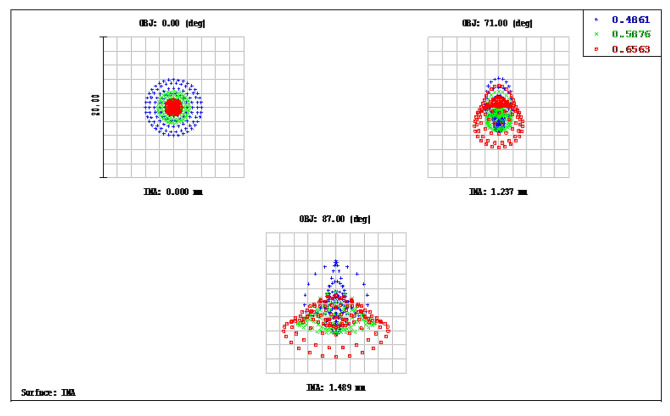
Spot diagram size of a miniaturized wide-angle fisheye lens using an OKP4HT aspherical lens; the figure shows the RMS spread of the images at three field angles of 0°, 71°, and 87°. The blue, green, and red dots represent the wavelengths of 0.4861, 0.5876, and 0.6563 μm, respectively.

**Figure 5:**
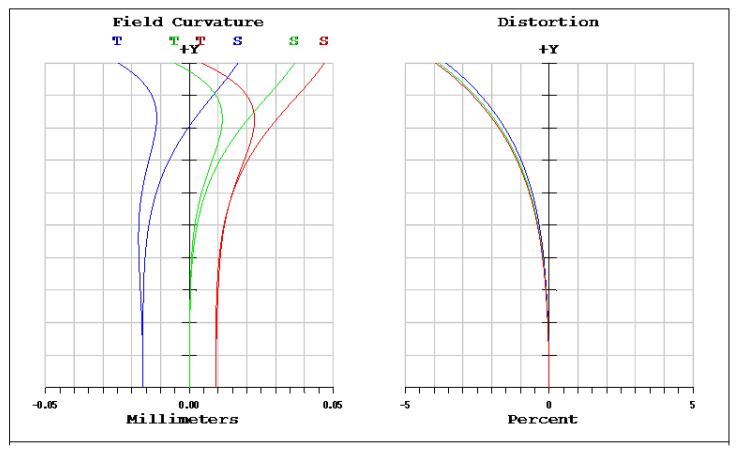
Field curvature (**left**) and distortion plot (**right**) of a miniaturized wide-angle fisheye lens using an OKP4HT aspherical lens.

**Figure 6:**
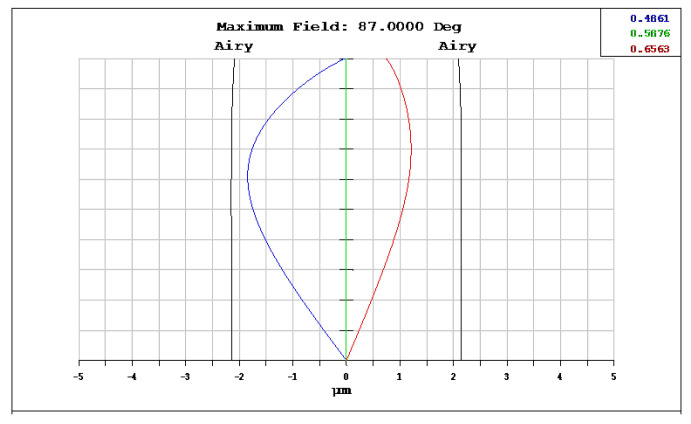
Lateral color of a miniaturized wide-angle fisheye lens combined with an OKP4HT aspherical lens. The blue, green, and red lines represent the wavelengths of 0.4861, 0.5876, and 0.6563 μm, respectively.

**Figure 7:**
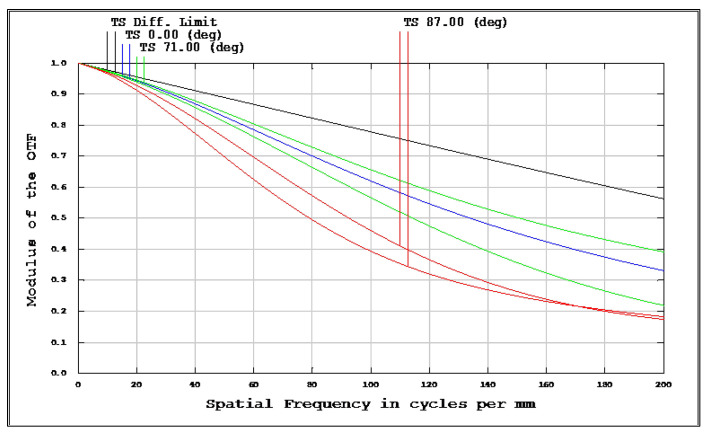
Modulation-transfer-function (MTF) curves of a miniaturized wide-angle fisheye lens combined with an OKP4HT aspherical lens. The blue, green, and red lines represent the tangential and sagittal (T = tangential; S = sagittal) directions for the 0°, 71°, and 87° cases, respectively.

**Figure 8:**
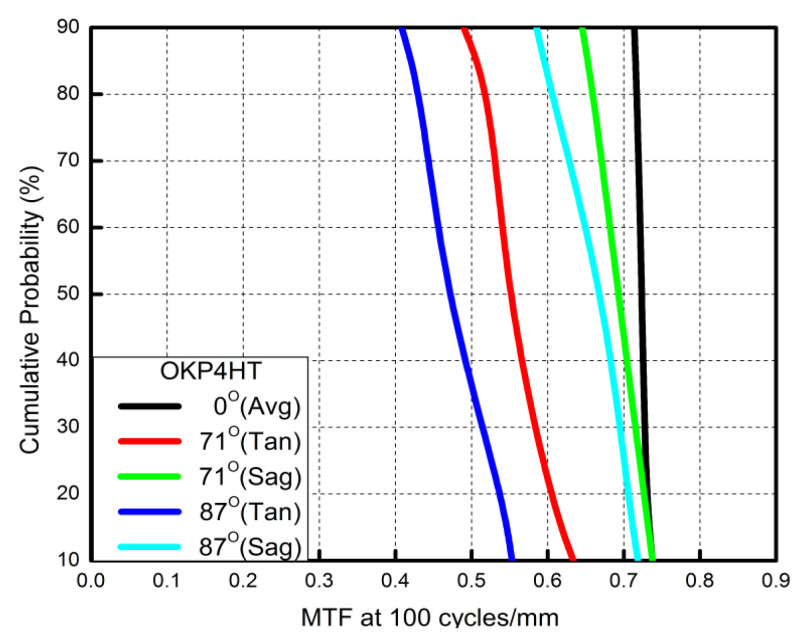
Cumulative probability vs. modulation transfer function (MTF) for a fisheye lens combined with an OKP4HT aspherical lens. For the cumulative probability of 90%, the tangential MTF values at the half FOV for the 0°, 71°, and 87°cases correspond to 0.72, 0.48, and 0.41, respectively.

The OKP4HT plastic material has the features of a high refractive index of 1.632 for thinner lenses and a low Abbe number of 23 for the correction of chromatic aberrations. We used the Zemax optical design software to simulate and optimize optical lens systems based on the DLS and genetic algorithms. Table 2 shows the lens data of a miniaturized wide-angle fisheye lens using an OKP4HT aspherical lens and four glass spherical lenses. It presents the radius, thickness, lens material, and lens diameter of each element. We selected the third and fourth surfaces as aspheric surfaces. First, the “cone” of the surface that needed to be changed into an aspheric surface was set as a variable and had to be optimized [30]. Second, the menu of “surface type” was set as “even asphere”. Then, we gradually set the fourth and higher-order coefficients as variables and ran the optimization until the result was significantly good. The optimized fourth-order aspherical coefficients were −0.003 for the third surface and 0.010 for the fourth surface. The optimized conic constant of the OKP4HT aspherical lens were −19.021 and −0.997 for the third and fourth surfaces, respectively. Our design configuration of the miniaturized wide-angle fisheye lens system is shown in Figure 3. There are five lens elements, including an aspheric lens (the second lens) and four glass spherical lenses. The results of the fisheye lens system design showed a total length of 15.0 mm, half-FOV of 87°, and EFL of 0.69 mm. The spot size diagram shows how light rays were focused on the ideal imaging surface. Figure 4 shows the spot diagrams of a wide-angle fisheye lens using one OKP4HT aspherical lens. A spot size of 6.11 μm for the off-axial angle was computed by the ZEMAX optics software. It indicated the RMS spread of the images at three field angles of 0° (on-axis), 71°, and 87°. The blue, green, and red dots represent wavelengths of 0.4861, 0.5876, and 0.6563 μm, respectively. The wavelength was set in the visible range of F, d, and C, which corresponded to 486.1, 587.6, and 656.3 nm, respectively. Optical distortion is one of the important quality assessment requirements. Usually optical distortion becomes large with the increasing of the FOV. In this work, the F-theta distortion was within ±3.5%, and the field curvature was within ±0.04 mm, as shown in Figure 5. The lateral color was within ±1.0 μm, as shown in Figure 6. The optical transfer function (OTF) is an important indicator of a lens performance. The OTF describes both the amplitude and the phase of a signal. In most cases, the modulus of the OTF can be expressed as MTF, which is often used as a mechanism for specifying the required image quality. The Zemax optical lens design software can compute the data of the geometric modulation transfer function as a function of field position. Figure 7 shows a plot of the MTF versus spatial frequency for three field positions. The spatial frequency is expressed in terms of cycles per millimeter. Different color lines represent different fields of view. The black line is a plot for the ideal lens system. The blue, green, and red lines represent tangential (abbreviated to T) and sagittal (abbreviated to S) directions for the 0°, 71°, and 87° cases, respectively. Figure 7 also shows a larger than 0.4 MTF value for the spatial frequency of 100 cycles/mm at the half-field angle of 87°. The maximum MTF value was 0.78 for the half-field angle of 0°. Figure 8 shows the design results of the tolerance analyses in different fields of view. Tolerance analysis was used to evaluate the production ability of the wide-angle fisheye lens; for this analysis, all parameters were assumed to have the same probability within plus and minus tolerances. We reviewed the data generated by the tolerance analysis and considered the tolerance budget. When necessary, the tolerance budget and the replications of the analysis were modified. For the cumulative probability of 90%, the tangential MTF values at the half FOV for the 0°, 71°, and 87° cases corresponded to 0.72, 0.48, and 0.41, respectively. This means that 90% of the MTF values in 100 trials were between 0.41 and 0.72.

### 3.2. Aspheric Lens Using Polymethyl Methacrylate Material

**Figure 9:**
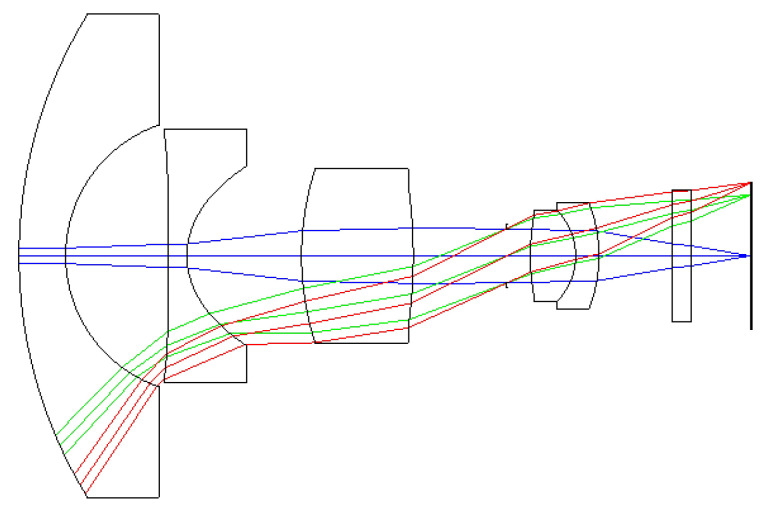
Design of a miniaturized wide-angle fisheye lens using a polymethyl methacrylate (PMMA) aspheric lens to replace a glass spherical lens.

**Figure 10:**
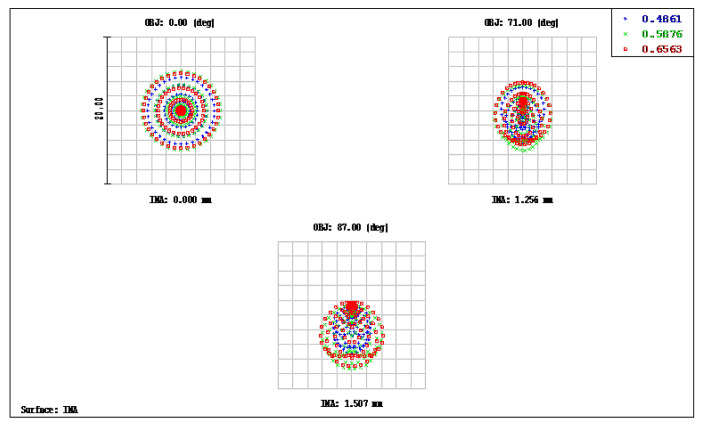
The spot size of a miniaturized wide-angle fisheye lens using a polymethyl methacrylate (PMMA) aspheric lens. The image also shows the RMS spread of the images at three field angles of 0°, 71°, and 87°. The blue, green, and red dots represent wavelengths of 0.4861, 0.5876, and 0.6563 μm, respectively.

**Figure 11:**
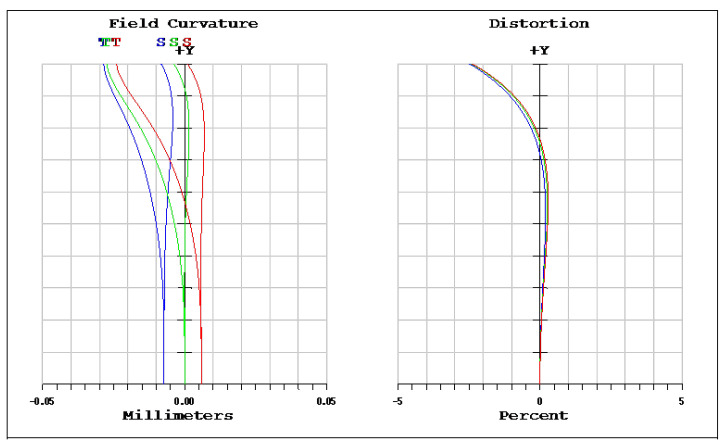
Field curvature (**left**) and distortion (**right**) of a miniaturized wide-angle fisheye lens using a polymethyl methacrylate (PMMA) aspheric lens.

**Figure 12:**
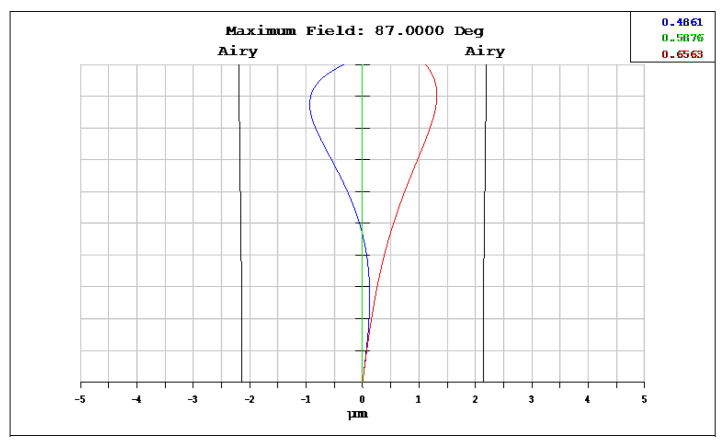
Lateral color of wide-angle fisheye lenses using a polymethyl methacrylate (PMMA) aspheric lens. The blue, green, and red lines represent the wavelengths of 0.4861, 0.5876, and 0.6563 μm.

**Figure 13:**
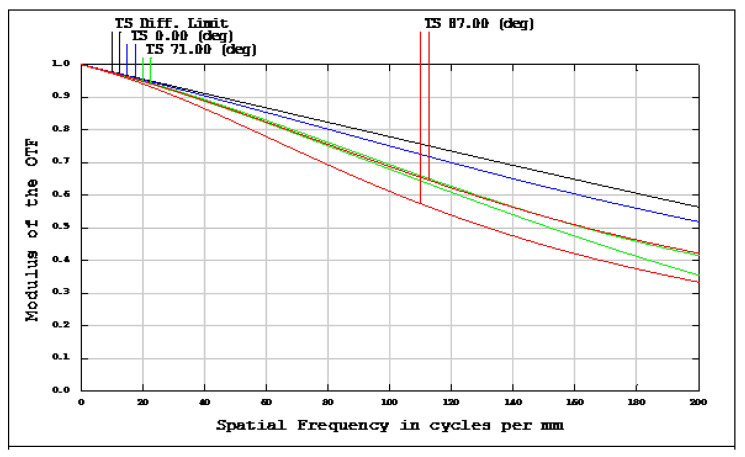
Modulation transfer function (MTF) of wide-angle fisheye lenses using a polymethyl methacrylate (PMMA) aspherical lens. The blue, green, and red lines represent the tangential and sagittal (T = tangential; S = sagittal) directions for the 0°, 71°, and 87°cases, respectively.

**Figure 14:**
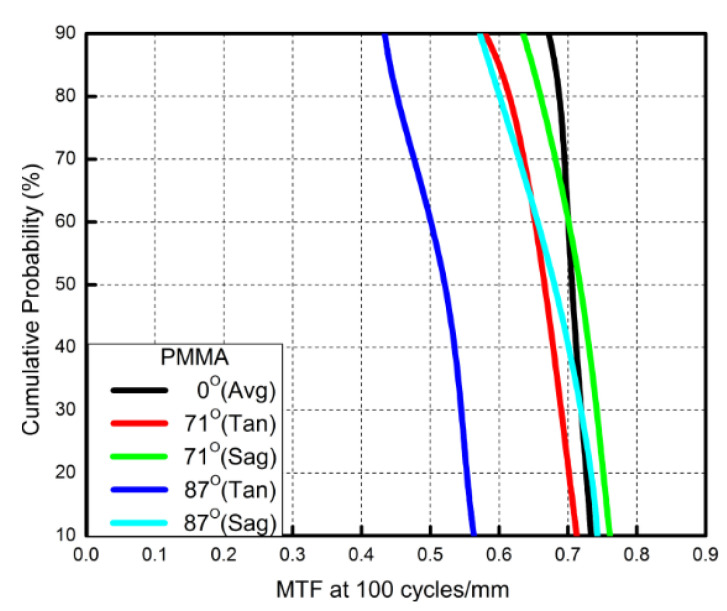
Cumulative probability vs. modulation transfer function (MTF) for fisheye lenses using a polymethyl methacrylate (PMMA) aspherical lens. For the cumulative probability of 90%, the tangential MTF values at the half field of view for the 0°, 71°and 87°cases correspond to 0.67, 0.58, and 0.43, respectively.

PMMA is known as plexiglass or acrylic glass. PMMA has a refractive index of 1.49 at 589.3 nm and a light transmittance of 92% in the visible spectrum [31]. PMMA absorbs UV light at wavelengths shorter than 400 nm and infra-red wavelengths higher than 2800 nm. These properties make it an excellent replacement for glass as an optical material. The data of a miniaturized wide-angle fisheye lens using a PMMA aspherical lens are presented in Table 3. The optimized fourth-order aspherical coefficients were −0.002 for the third surface and 0.005 for the fourth surface. The optimized conic constant of the OKP4HT aspherical lens was −20.008 and −0.749 for the third and fourth surfaces, respectively. In this study, the configuration of a miniaturized wide-angle fisheye lens system with an aspherical lens is shown in Figure 9. It consisted of five lens elements including a PMMA aspheric lens. Our design can be improved using aspheric surfaces without adding additional lenses. Since adding more aspheric surfaces will increase the manufacturing cost of the components and the tolerances, it is best to keep the number and degree of aspheric surfaces to a minimum. The fisheye lens system design results showed a total length of 14.5 mm, half FOV of 87°, and EFL of 0.89 mm. The spot size was 2.185 μm, as shown in Figure 10. The distortion was within ±3.0%, and the field curvature was within ±0.02 mm (Figure 11). The lateral color was within ±1.0 μm, as shown in Figure 12. The MTF plot is shown in Figure 13; according to it, the MTF value was larger than 0.6 for the spatial frequency of 100 cycles/mm at the field angle of 174°. The MTF value of this design was found to be higher than that of the previous one. The results of the tolerance analysis at different fields of view are shown in Figure 14. For the cumulative probability of 90%, the tangential MTF values at the half field of view of the 0°, 71°, and 87°cases corresponded to 0.67, 0.58, and 0.43, respectively. In other words, 90% of the 100 trials showed MTF values between 0.43 and 0.67. As described above, Table 2 and Table 3 present the lens design data of the miniaturized wide-angle fisheye lens using an OKP4HT and PMMA aspherical lens combined with four glass spherical lenses, respectively. In the two design cases, the materials of four glass lenses were the same, but the materials and the diameter-to-thickness ratio of the second plastic aspheric lens were different.

Traditionally, computer-optimized optical design uses a numerical merit function to represent the optical performance of the simulated system. The traditional design method involves maximizing the nominal performance of the design and then adding the manufacturing tolerance to the nominal parameters as a separate step, so that the resulting system can still meet the specification requirements during manufacturing. Moore [32] verified that optimization using the hybrid merit function produces different forms of designs that may have lower nominal performance but higher as-built performance. However, we applied different lens material combinations to add an aspherical polymetric lens by a deep learning algorithm. We observed that the performance of a miniaturized wide-angle fisheye lens using one PMMA aspherical lens and four spherical lenses was better than that of a fisheye lens using an OKP4HT aspherical lens design, in terms of RMS spot radius, field curvature, distortion, and MTF values.

## 4. Conclusions

We designed a miniaturized wide-angle fisheye lens that couples the ZEMAX software with a deep learning algorithm to achieve the design requirements. In this work, we proposed two types of miniaturized wide-angle fisheye lens optimization designs using four spherical lenses and one aspheric lens. To meet the design specifications for the miniaturized wide-angle fisheye lens system, the ZEMAX software was used with merit functions for optimizing the MTF performance. The design results of the two cases showed a full viewing angle of 174°, a total length less than 15 mm, a spot size less than 6 μm, lateral color within ±1 μm, field curvature within ±0.02 mm, and F-theta distortion within ±3.5%. The lens designers must figure out which lens tolerances they can apply and predict the production yields. For the tolerance analysis, the MTF value was larger than 0.4 at the spatial frequency of 100 cycles/mm. We compared the design results of two miniaturized wide-angle fisheye lenses. The fisheye lens system combined with a PMMA aspherical lens was found to perform better than the system with an OKP4HT aspherical lens, despite also the latter meeting the design specifications. These design results show that deep learning can help design novel wide-angle fisheye lenses. In the future, we can introduce metalenses to obtain more compact ultra-wide-angle fisheye lenses.

## Figures and Tables

**Table 1:** Design specifications of a miniaturized wide-angle fisheye lens.

Items	Design Requirements
F/#	3.0
FOV(°)	>170°
EFL (mm)	>0.6 mm
MTF (%) at 100 lp/mm	>30%
Field curvature (mm)	<0.5 mm
Spot size (μm)	<5 μm
Total length (mm)	<20 mm
F-θ distortion (%)	<5%
Lateral Color (μm)	<2 μm

**Table 2:** Lens data of a miniaturized wide-angle fisheye lens using an OKP4HT aspherical lens and four glass spherical lenses.

Surface No./Type		Radius (mm)	Thickness (mm)	Material	Diameter (mm)
1	STANDARD	0.115	1.232	LASF18A	10.886
2	STANDARD	0.392	2.269		5.062
3	ASPHERICAL	0.067	0.544	OKP4HT	4.918
4	ASPHERICAL	0.587	2.475		3.660
5	STANDARD	0.174	0.889	SF6	4.078
6	STANDARD	−10.127	2.855		4.026
7	APERTURE STOP	Infinity	0.576		1.084
8	STANDARD	0.323	1.109	N-AK34	1.842
9	STANDARD	−10.684	0.368	SF66	1.982
10	STANDARD	−10.217	1.500		2.220

**Table 3:** Lens data of a miniaturized fisheye lens using a polymethyl methacrylate (PMMA) aspherical lens.

Surface No./Type		Radius (mm)	Thickness (mm)	Material	Diameter (mm)
1	STANDARD	0.106	0.956	LASF18A	9.914
2	STANDARD	0.352	2.099		5.368
3	ASPHERICAL	0.006	0.387	PMMA	5.176
4	ASPHERICAL	0.628	2.350		3.682
5	STANDARD	0.172	2.291	SF6	3.568
6	STANDARD	−0.096	1.919		2.952
7	APERTURE STOP	Infinity	0.483		1.112
8	STANDARD	0.214	0.928	N-LAK34	1.682
9	STANDARD	−0.754	0.477	SF66	1.854
10	STANDARD	−0.330	1.500		2.716

## Data Availability

Not applicable.
